# Precise levelling in crossing river over 5 km using total station and GNSS

**DOI:** 10.1038/s41598-021-86929-1

**Published:** 2021-04-05

**Authors:** DingLiang Yang, JinGui Zou

**Affiliations:** 1 School of Geodesy and Geomatics, Wuhan University, Wuhan, China; 2 Beijing Key Laboratory of Urban Spatial Information Engineering, Beijing, China

**Keywords:** Engineering, Civil engineering

## Abstract

The trigonometric levelling using the simultaneous reciprocal method has been proved to meet the precision of second order levelling. But this method is invalid once the distance of river crossing is beyond 3.5 km due to the difficulty of target recognition at such a long distance. To expand the available range of this method, this paper focuses on solving the target aiming and distance observation over a long distance. A modular LED 5-prism (modified Leica GPR1 reflector) as an illuminated target instead of the common prism is introduced, and we adopt the sub-pixel image processing technique to recognize the center of the target image pictured by image assisted total station (Leica Nova TM50 I equipped with a coaxial camera). Based on the principle of precise trigonometric levelling, this paper utilizes two image assisted total stations using image processing technique to perform simultaneous reciprocal for zenith angle measurement and GNSS static measurement for slope distance measurement to determine the height difference of either river bank. And long-distance precise river-crossing levelling can be realized based on the mentioned above. Besides, it is successful to apply in the experiment of Fuzhou Bridge spanning 6.3 km in China. The result shows the standard deviation is ± 0.76 mm/km that is compatible with the precision of second order levelling has.

## Introduction

Connecting different elevation systems on either riverbank is the guarantee of vertical control for bridge construction. Common methods for determining height difference include geometric levelling, trigonometric levelling, GNSS levelling and hydrostatic level. Geometric levelling is the earliest but most mature method to achieve height transfer with high accuracy^1^. Benefiting from the rigid regulation of operating, geometric levelling is the first choice used in precise levelling. However, the restricted requirements of the short distance of sight length (30 m to 50 m) in precise levelling and the equal back- and fore-sight length hardly make geometric levelling for impassable terrains such as valleys, fjords and rivers. Zeiss Oberkochen developed a specialized river-crossing instrument for river-crossing levelling of medium length (1 km to 2 km;^2,3^). And the accuracy of using the Zeiss levels in river-crossing levelling is compatible with the precise levelling has^4,5^. But it needs a huge surveyor’s beacon and demands rich experienced surveyors. Other methods for determining height difference across rivers include hydrostatic levelling and hydromantic levelling. Though those methods are accurate enough but extremely expensive and their accuracy will decrease with the increasing spanning distance^6^. GNSS levelling is a fast way to get the height difference of two points by transforming between GNSS geodetic height and normal height. But its accuracy only meets third order levelling which can not be used for precise levelling. Finally, trigonometric levelling by precise robotic total stations can be utilized as well for river-crossing levelling.

Trigonometric levelling (TL), merely an optional method to quickly determine the height difference of points, is not substituted for the precise levelling before the appearance of robotic total stations. Since the invention of the robotic total station, many scholars have investigated the theories and implementations of precise trigonometric levelling^7,8^. Zhenglu^7^ analyzed the errors of TL and derived the rigid formula of precise trigonometric levelling (PTL), in theory, to replace the first order levelling. Then the leap-frog method and simultaneous-reciprocal observation method were respectively put into practice for improving the accuracy of TL to achieve PTL^9,10^. Zou^11^ combined the leap-frog method and simultaneous-reciprocal observation method and developed a motorized PTL system (MPTL) used in tough terrains. And the standard deviations of those applications all meet the accuracy of second-order levelling. But the average length of observation sides of these applications is at a distance of a few hundred meters.

Nowadays, the length of most modern river-crossing bridges is beyond 1 km that makes it difficult to automatically point the target using a total station. The performance of automatic target recognition (ATR) of a total station decreases for the large distances due to the weakened intensity of the reflected laser beam of robotic total stations. And the common trigonometric levelling method is hard to apply in river-crossing levelling. Some researchers mount an external camera on the eyepiece of the total station for optical automatic target recognition^12^ and usefully in bridge vibration monitoring^13–16^. Fortunately, several types of total stations have a built-in coaxial camera such as Leica TM50 I and MS50, and these total stations can take images and store images on their memory or SD card. Therefore, in this paper, based on the principle of precise trigonometric levelling, we proposed a new scheme for long-distance rive-crossing levelling by two total stations built-in coaxial cameras (image-assisted total station) and GNSS. The image-assisted total station is used for zenith angle observation and GNSS is used for slope distance measurement. For realizing the automatic recognition of a target beyond the survey range of the total station, a special modular 5-prism with LEDs built-in as the recognition target is made and the image processing technique and highly accurate image-assisted total station to automatically obtain precise zenith angular measurements are applied. And the large distances across the river are measured by GNSS static network measurement. Then, a survey routine is designed for avoiding the measurement of the instrument's height and target’s height. Finally, a test of Fuzhou Bridge in China which has 6.3 km is tested.

## Method of river-crossing levelling

### Principle and error analysis of Trigonometric levelling

The trigonometric levelling method uses the slope distance $S$ and zenith angle $\alpha$ to determine the height difference of two points. When two points are far apart, the effects of refraction and earth curvature need to be considered. And the formula for trigonometric levelling is as follows^2^:

1 $$h_{1,2} = S_{{{1},{2}}} \cos \alpha_{1,2} + \frac{1 - K}{{2R}}\left( {S_{1,2} \sin \alpha_{1,2} } \right)^{2} + i_{1} - v_{2}$$

where ${i}_{1}$ is the height of the instrument and ${v}_{2}$ is the height of the target, K is the atmospheric refraction coefficient and $\mathrm{R}$ is the radius of the local sphere in kilometers.

Here, the standard deviation of zenith angle, distance and uncertainties of the coefficient of atmospheric refraction are denoted by ${\sigma }_{\alpha }$, ${\sigma }_{s}$ and ${\sigma }_{k}$. The standard deviations of measurement of the instrument’s height and target’s height are denoted by ${\sigma }_{i}$ and ${\sigma }_{v}$. The estimated variance of ${h}_{\mathrm{1,2}}$ is expressed in formula (Eq. 2) which is derived from formula (Eq. 1) based on the law of variance propagation^7^.

2 $$\sigma_{{h_{1,2} }}^{2} = \cos \alpha^{2}_{1,2} \cdot \sigma_{S}^{2} + \left( {\frac{{S\sin \alpha_{1,2} }}{{\rho^{\prime\prime}}}} \right)^{2} \sigma_{\alpha }^{2} + \left( {\frac{{S^{2} \sin \alpha^{2}_{1,2} }}{2R}} \right)^{2} \sigma_{k}^{2} + \sigma_{i}^{2} + \sigma_{v}^{2}.$$

**Figure 1:**
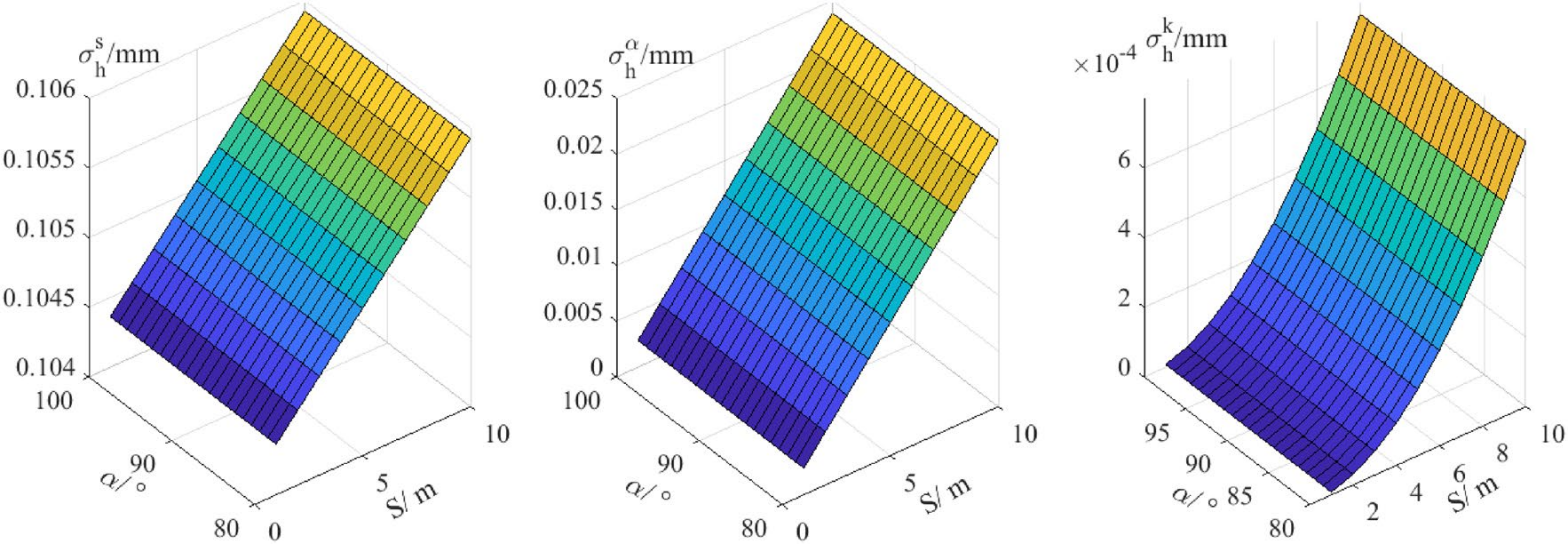
Errors analysis of trigonometric levelling in 10 m.

**Figure 2:**
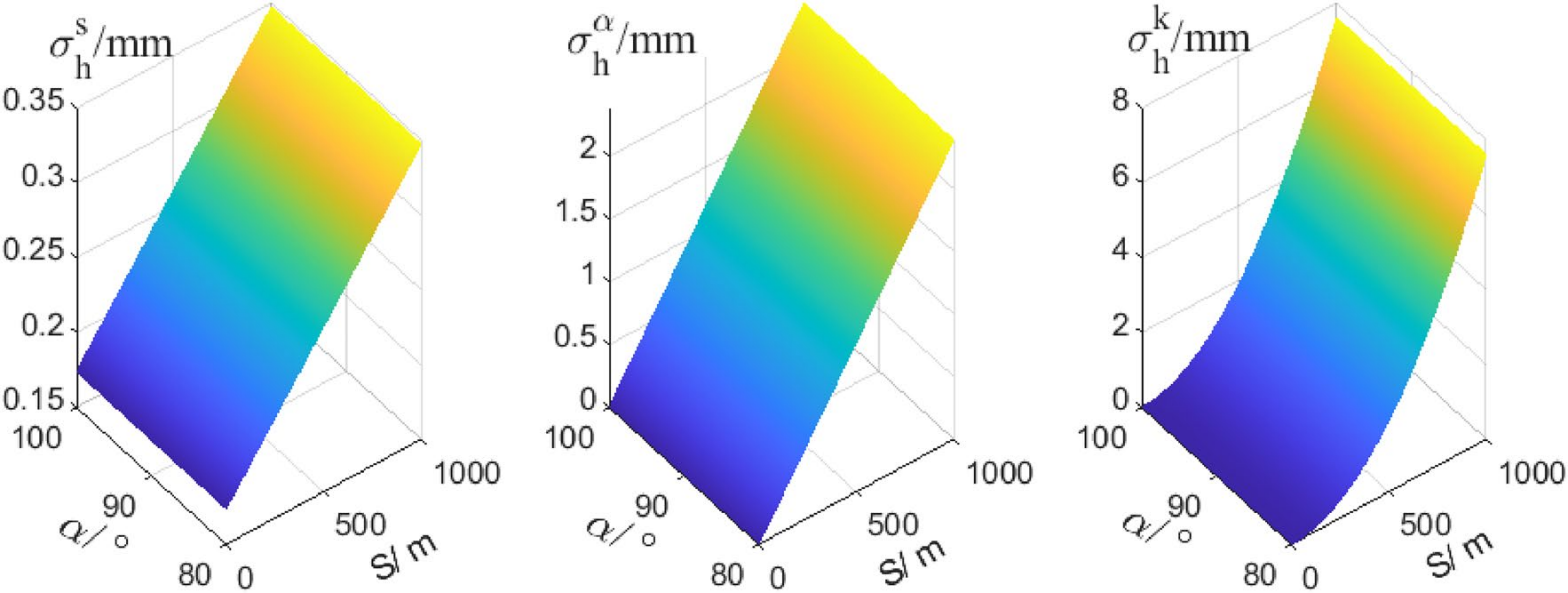
Errors analysis of trigonometric levelling in 1000 m.

The magnitude of the standard deviations of ${\sigma }_{i}$ and ${\sigma }_{v}$ is ± 0.3 mm using the method proposed by^17–19^. The standard deviations of zenith angles and distance of robotic total station are up to 0.5 arcsec and 1 mm + 1 ppm respectively. Traditionally, the empirical refraction value of 0.13 is used for the refraction effects in unidirectional zenith angle observation^18^. The uncertainties in the coefficient of refraction are taken as ± 0.1 for zenith angles observations. Here, the effects of ${\sigma }_{\alpha }$, ${\sigma }_{s}$ and ${\sigma }_{k}$ on the precision of trigonometric levelling are denoted as ${\sigma }_{h}^{\alpha }$, ${\sigma }_{h}^{s}$ and ${\sigma }_{h}^{k}$ respectively. With different distances and zenith angles, the numerical values of ${\sigma }_{h}^{\alpha }$, ${\sigma }_{h}^{s}$ and ${\sigma }_{h}^{k}$ are shown in Figs. 1 and 2.

Figure 1 shows in a short distance of 10 m, with the zenith angle measuring from 80 to 100 degrees, the effect of measuring distance error (${\sigma }_{h}^{s}$) plays a leading role to the precise of trigonometric levelling and the maximum of ${\sigma }_{h}^{s}$ is up to 0.1 mm. Compared the maximum of ${\sigma }_{h}^{s}$ with ${\sigma }_{h}^{\alpha }$ and ${\sigma }_{h}^{k}$ in the range of 10 m, the effects of measuring angle error (${\sigma }_{h}^{\alpha }$) and atmospheric refraction uncertainty (${\sigma }_{h}^{k}$) to precision of trigonometric levelling can be ignored whose values are all less than 0.03 mm. It follows that when the measuring distance is within 10 m, the term of atmospheric refraction in formula (Eq. 1) can be ignored.

Figure 2 shows with the increasing distance the effect of atmosphere refraction uncertainty increases obviously and can be up to 8 mm in the distance of 1000 m. The effect of measuring distance error very slowly changes with the increasing distance. Compared the effects of ${\sigma }_{h}^{k}$ with ${\sigma }_{h}^{\alpha }$ and ${\sigma }_{h}^{s}$ to the precision of trigonometric levelling with the increasing measuring distance, the effect of atmospheric refraction uncertainty plays the leading role, followed by that of measuring angle error. It follows that when the measuring distance the influence of atmospheric refraction can’t be ignored and must take some appropriate methods to reduce the influence of atmospheric refraction.

### Implement trigonometric levelling in river-crossing levelling

**Figure 3:**
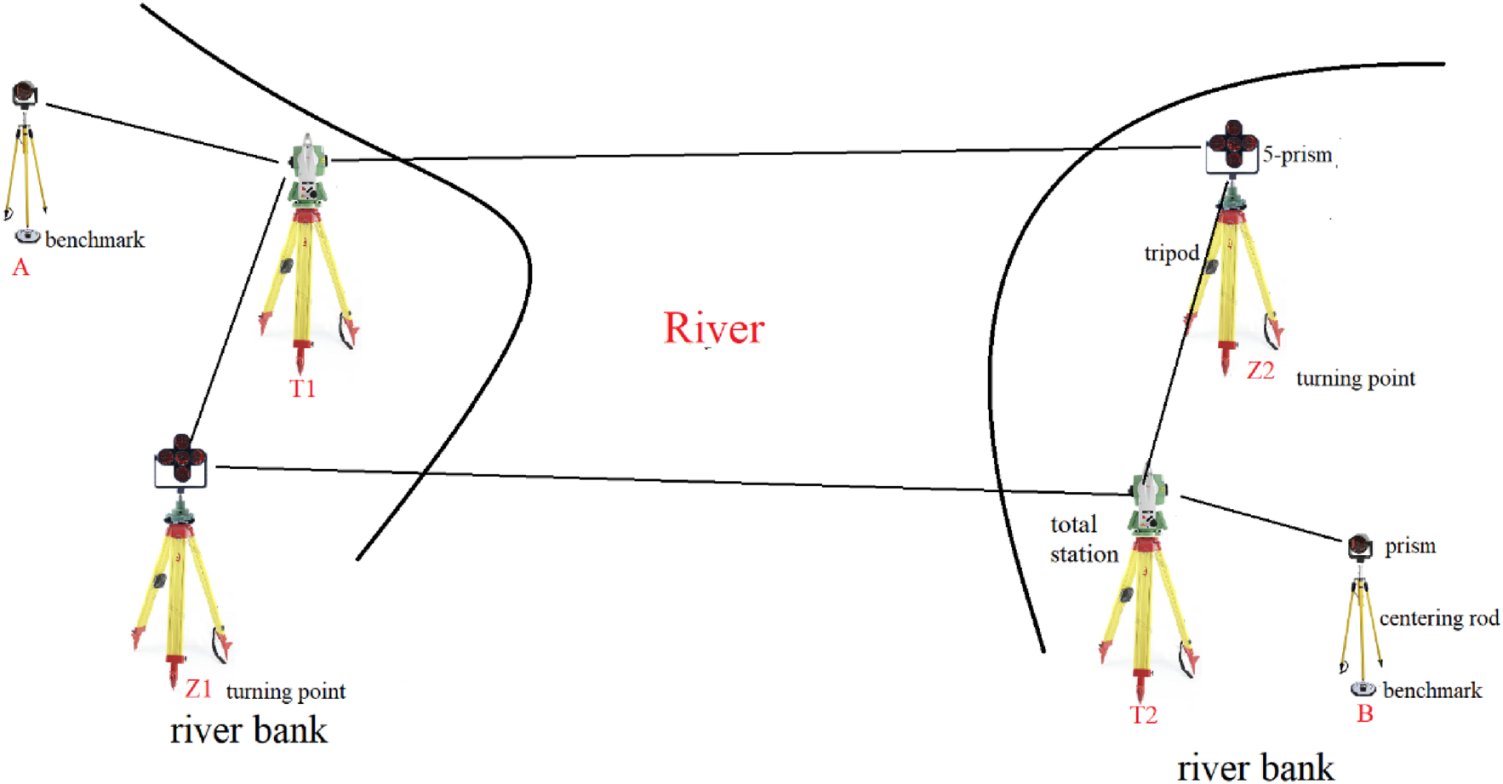
The surveying line of trigonometric levelling between two benchmarks on either side of the river.

The history of river-crossing levelling is over three decades. The main methods to transfer the height across the river are geometric levelling and trigonometric levelling. The accuracy of determining the height difference of both of them meets that of second-order levelling has in a distance above hundreds of meters^4,9,10^. And the effect of refraction is the main factor to influence the precision of river-crossing levelling^19^. In this paper, we use two robotic total stations to perform river-crossing levelling and Fig. 3 shows the river-crossing surveying line of trigonometric levelling method proposed in this paper.

In Fig. 3, it is required that put the same type prism and centering rod with graduation and circular level bubble at benchmark A and B respectively and adjust the two centering rods to the same height so that the height of the target at benchmark A is equal to that of benchmark B. Besides, it is also required that the distance from a total station at point T1 to the benchmark A or the turning point Z1 is no more than 10 m and a total station at point T2 is also within 10 m far from the benchmark B or the point Z2. To reduce the effect of vapor on the precision of trigonometric levelling, the points Z1, T1, Z2 and T2 are all above the river surface 5 to 10 m. The zenith angle observation from T1 to Z2 is approximately simultaneous performed that of from T2 to Z1. As the intensity of the laser beam is weakened by the larger distances across the rives, the GNSS is used to measure the river-crossing distance. At last, 5-prism with LEDs installed specially designed are used as pointing target and the high accuracy robotic total stations built-in coaxial cameras are selected to ensure the accuracy of angular observation during the river-crossing levelling.

The height difference between benchmark A and B (${h}_{A,B}$) on either side of the river is divided into three parts (${h}_{A,Z1}$, ${h}_{Z1,Z2}$ and ${h}_{Z2,B}$). The height difference of benchmark A and point Z1 is denoted by ${h}_{A,Z1}$, the height difference of benchmark Z1 and point Z2 and the height difference of point Z2 and benchmark B are denoted by ${h}_{Z1,Z2}$ and ${h}_{Z2,B}$ respectively. Because the distance from T1 to benchmark A or Z1 is within 10 m, the term of refraction on formula (Eq. 1) can be omitted, so the formula of ${h}_{A,Z1}$ is expressed by:

3 $$h_{A,Z1} = h_{T1,Z1} - h_{T1,A} = \left( {S_{T1,Z1} \cos \alpha_{T1,Z1} + i_{T1} - v_{Z1} } \right) - (S_{T1,A} \cos \alpha_{T1,A} + i_{T1} - v_{A} )$$

where $S$ represents the slope distance from a total station to the target, $\alpha$ denotes the zenith angle from a total station to a target, $i$ represents the height of a total station and $v$ represents the height of a target.

In Eq. (3), it can be seen the term of total station height can be eliminated when the total station is put among the targets. Therefore, the final equation of computing the height difference ${h}_{A,Z1}$ is expressed by:

4 $$h_{A,Z1} = h_{T1,Z1} - h_{T1,A} = S_{T1,Z1} \cos \alpha_{T1,Z1} - v_{Z1} - S_{T1,A} \cos \alpha_{T1,A} + v_{A}.$$

The distance from T2 to benchmark B or Z2 is also within 10 m and the formula of ${h}_{Z2,B}$ can be derived from Eq. (4).

5 $$h_{Z2,B} = h_{T2,B} - h_{T2,Z2} = S_{T2,B} \cos \alpha_{T2,B} - v_{B} - S_{T2,Z2} \cos \alpha_{T2,Z2} { + }v_{Z2} .$$

To estimate the height difference of points Z1 and Z2 (${h}_{Z1,Z2}$) which crosses the river, we need to perform the river-crossing trigonometric levelling. On account of the wide width of the river, the effects of refraction and earth curvature to trigonometric levelling can not be omitted. The coefficient of refraction can be estimated in trigonometric traverse but its values are various at different altitudes and different periods of 1 day^19^. So, it is difficult to build an approximately common mathematic model to calculate the coefficient of refraction. But several applications show that the coefficient of refraction remains stable in cloudy weather or the evening. These valuable conclusions can guide us to choose a suitable period to perform river-crossing levelling using the trigonometric levelling method. Additionally, some researchers proposed that if the simultaneous reciprocal observation is applied in trigonometric levelling, the effect of refraction will be highly weakened and the accuracy of the result meets that of the precise levelling has. However, the rigid reciprocal observation at the same time hardly realized and in practice, we develop a data collection software to complete the whole reciprocal observation in a short time and to ensure the observation lines by total stations on either side of the river be nearly equal not only the measuring distance but also the same condition of observation, e.g. temperature and vegetation, vapor and refraction.

In this paper, we adopt the parallelogram network in Fig. 3 (Z1–T1–Z2–T2–Z1) to achieve the approximately simultaneous reciprocal observation by robotic two total stations on either side of the river. Thus, the height difference of Z1 and Z2 is measured with two different surveying lines, one is from Z1 to T1 to Z2, another is from Z1 to T2 to Z2. And we take the arithmetic average of the height differences measured by the two surveying lines respectively as the final height difference of Z1 and Z2. The formula of calculation ${h}_{Z1,Z2}$ measured by a total station at T1 is expressed by:

6 $$h_{{_{Z1,Z2} }}^{T1} = h_{T1,Z2} - h_{T1,Z1}.$$

In Eq. (6), the term ${h}_{T1,Z2}$ is computed by the height difference of Z1 and T1 (${h}_{T1,Z1}$) and the height difference of T1 and Z2 (${h}_{T1,Z2}$). As the distance from Z1 to T1 is no more than 10 m and the distance from T1 to Z2 is large, we ignore the effects of atmospheric refraction and earth curvature to ${h}_{T1,Z1}$ and consider that to ${h}_{T1,Z2}$. Therefore, the formulas of ${h}_{T1,Z1}$ and ${h}_{T1,Z2}$ are as follows.

7 $$\left\{ \begin{gathered} h_{T1,Z1} = S_{{_{T1,Z1} }} \cos_{T1,Z1} + i_{T1} - v_{Z1} \hfill \\ h_{T1,Z2} = S_{{_{T1,Z2} }} \cos_{T1,Z2} + i_{T1} - v_{Z2} + \frac{{1 - K_{{_{T1,Z2} }} }}{2R}\left( {S_{{_{T1,Z2} }} \sin_{T1,Z2} } \right)^{2} \hfill \\ \end{gathered} \right.$$

where K represents the coefficient of atmospheric refraction and R denotes the mean radius of the earth in km.

Substitute Eq. (7) into Eq. (6), the height difference of Z1 and Z2 measured by a total station at point T1is shown as:

8 $$h_{Z1,Z2}^{T1} = S_{T1,Z2} \cos \alpha_{T1,Z2} - v_{Z2} - S_{T1,Z1} \cos \alpha_{T1,Z1} + v_{Z1} + \frac{{1 - K_{T1,Z2} }}{2R}\left( {S_{{_{T1,Z2} }} \sin_{T1,Z2} } \right)^{2} .$$

The calculation progress of height difference of Z1 and Z2 determined by a total station at T2 is the same as that by a total station at T1. Therefore, the formula of ${h}_{Z1,Z2}$ by a total station at T2 is expressed by:

9 $$h_{Z1,Z2}^{T2} = S_{T2,Z2} \cos \alpha_{T2,Z2} - v_{Z2} - S_{T2,Z1} \cos \alpha_{T2,Z1} + v_{Z1} - \frac{{1 - K_{T2,Z1} }}{2R}\left( {S_{T2,Z1} \sin_{T2,Z1} } \right)^{2}.$$

According to Eqs. (8) and (9), the final height difference of Z1 and Z2 is the arithmetic average of ${h}_{Z1,Z2}^{T1}$ and ${h}_{Z1,Z2}^{T2}$.

10 $$h_{Z1,Z2} = \frac{1}{2}\left( {S_{T2,Z2} \cos \alpha_{T2,Z2} - S_{T1,Z1} \cos \alpha_{T1,Z1} + S_{T1,Z2} \cos \alpha_{T1,Z2} - S_{T2,Z1} \cos \alpha_{T2,Z1} } \right) - v_{Z2} + v_{Z1} + M$$

where $$M = \frac{{1 - K_{T1,Z2} }}{4R}\left( {S_{T1,Z2} \sin_{T1,Z2} } \right)^{2} - \frac{{1 - K_{T2,Z1} }}{4R}\left( {S_{T2,Z1} \sin_{T2,Z1} } \right)^{2}$$.

Then the height difference of benchmarks A and B is derived by Eqs. (4), (5) and (10).

11 $$\begin{gathered} h_{A,B} = \left( {S_{T2,B} \cos \alpha_{T2,B} - S_{T1,A} \cos \alpha_{T1,A} } \right) + \frac{1}{2}\left( {S_{T1,Z1} \cos \alpha_{T1,Z1} - S_{T2,Z2} \cos \alpha_{T2,Z2} } \right) \hfill \\ \begin{array}{*{20}c} {} & {} & {} \\ \end{array} + \frac{1}{2}\left( {S_{T1,Z2} \cos \alpha_{T1,Z2} - S_{T2,Z1} \cos \alpha_{T2,Z1} } \right) + (v_{A} - v_{B} {) + }M \hfill \\ \end{gathered} .$$

In Eq. (11), ${v}_{A}$ will be equal to ${v}_{B}$ if we put the same centering rods with graduation and prisms on benchmarks. In practice, the sight from T1 to Z2 is parallel to that from T2 to Z1, thus the horizontal distance from T1 to Z2 is approximately equal to that from T2 to Z1. And all the instruments on either side of the river are 5 to 10 m above the river surface for reducing the effects of vapor and vegetation and getting stable atmosphere refraction. Besides, the vertical angle from the total station to the target on the opposite river bank is no more than 1 degree for maintaining the same condition of reciprocal observation sight. Based on the mentioned above the coefficient of atmosphere refractions of reciprocal observations are approximately equal, the term of atmospheric refraction (M) in Eq. (11) can be eliminated by simultaneous reciprocal observation. Therefore, in practice, the calculation formula of the height difference of benchmarks A and B is as follows.

12 $$\begin{gathered} h_{A,B} = \left( {S_{T2,B} \cos \alpha_{T2,B} - S_{T1,A} \cos \alpha_{T1,A} } \right) + \frac{1}{2}\left( {S_{T1,Z1} \cos \alpha_{T1,Z1} - S_{T2,Z2} \cos \alpha_{T2,Z2} } \right) \hfill \\ \begin{array}{*{20}c} {} & {} & {} \\ \end{array} + \frac{1}{2}\left( {S_{T1,Z2} \cos \alpha_{T1,Z2} - S_{T2,Z1} \cos \alpha_{T2,Z1} } \right) \hfill \\ \end{gathered}.$$

**Figure 4:**
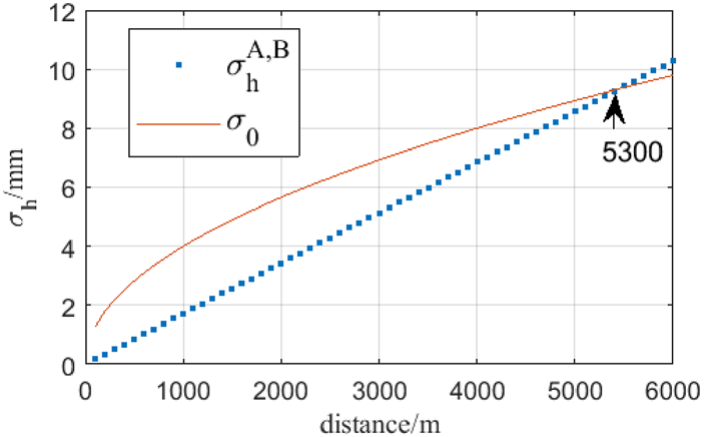
The precision estimation of simultaneous reciprocal trigonometric levelling.

It is obvious that in Eq. (12), the heights of instruments or targets are all removed, that’s to say, using the method we proposed is no necessary to measure instruments’ height and targets’ height. And only the terms of angular measurement and range measurement are left. Here, the standard deviations of distance, zenith angles are denoted by ${\sigma }_{s}$, ${\sigma }_{\alpha }$. Currently, the standard deviations of zenith angles and distance of robotic total station are up to 0.5 s and 1 mm + 1 ppm respectively. Given that the length of short distance (${S}_{T2,B}$, ${S}_{T1,A}$, ${S}_{T1,Z1}$, and ${S}_{T2,Z2}$) is 10 m, the long-range across the river is from 100 to 6000 m and the zenith angle is 80°. Take the derivation of Eq. (12) and use the law of variance propagation, the estimated variance of ${\sigma }_{h}^{A,B}$ is shown in Fig. 4.

In Fig. 4, ${\sigma }_{0}$ denotes the standard deviation of the second-order levelling which is computed by $4\sqrt{L}$ (L is horizontal distance in km). In theory, the precision of the simultaneous reciprocal trigonometric with high accuracy angular measurement can meet that of the second-order levelling has in a unidirectional distance above 5000 m.

## Distance and angle measurement

In the long-distance trigonometric levelling, the zenith angle observations and slope distance measurements are measured in multi-observation sets (the measurements both in the face left and face right called one observation set). The function of automatic target recognition (ATR) of robotic total station and self-developed data collection software is used in the crossing-river/valley trigonometric levelling to fast complete all the reciprocal observations simultaneously in few minutes^11^. However, the ranging of ATR of the robotic total station is determined by the reflection intensity of infrared light and the reflection intensity is influenced by the measuring distance and the weather condition^20^. Thus, the range of ATR is sometimes not sufficient with the requirement of engineering. Serval experiments show the distance difference of slope distance measured of two points by a total station and the baseline length of the same two points measured by GNSS is up to 7 mm^21,22^.

In the case of ATR useless, several researchers have realized the optical target recognition, not only the prism but also the ceramic ball, reflector plate, and lamp, by mounting the external cameras on the eyepiece of a total station^13^. Currently, several robotic total stations, such as the Leica TM50 I and the Leica MS50, have inside equipped with a telescope camera and a wide-angle camera and they can show images on the screen and store the image in its memory or SD card. And we can use the telescope camera of the total station to take the remote target image and image processing technique to achieve the remote target recognition.

### Distance measurement

**Figure 5:**
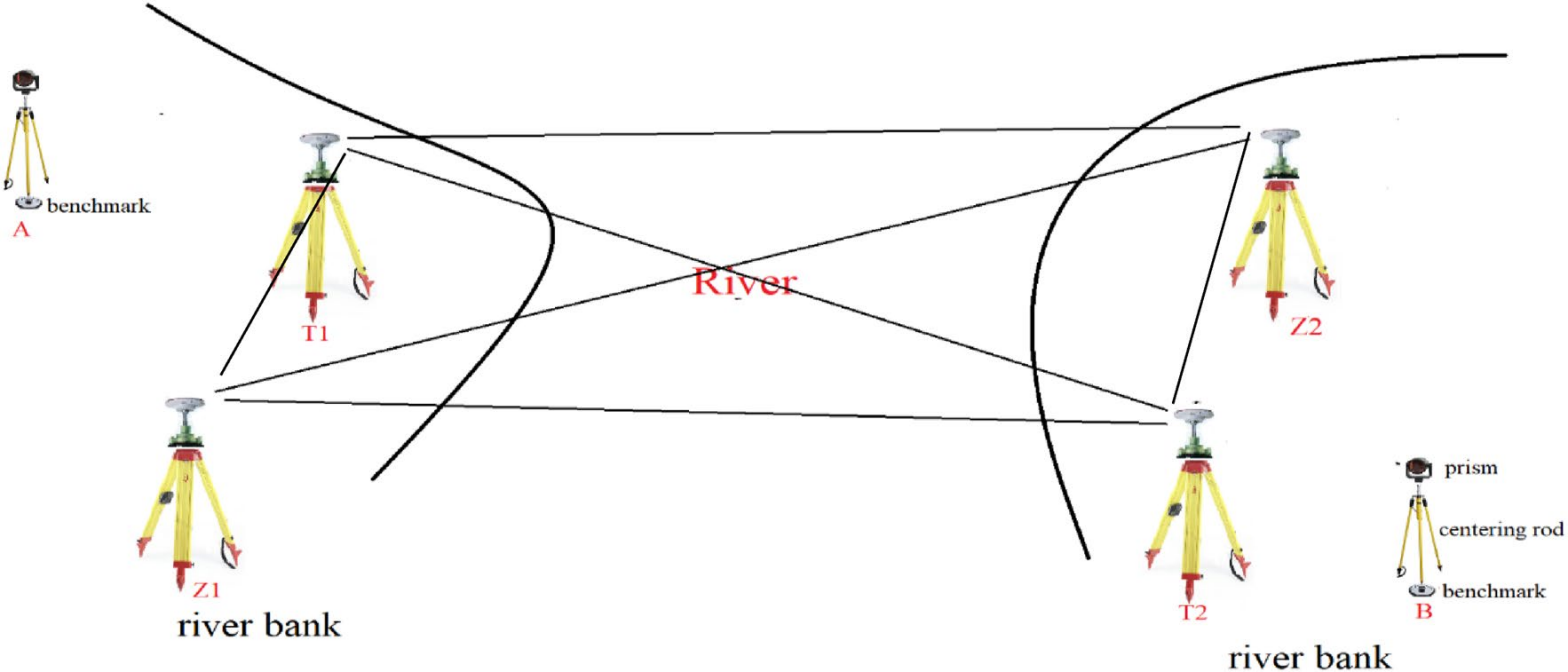
The river-crossing distance measurement using the static GNSS method.

**Figure 6:**
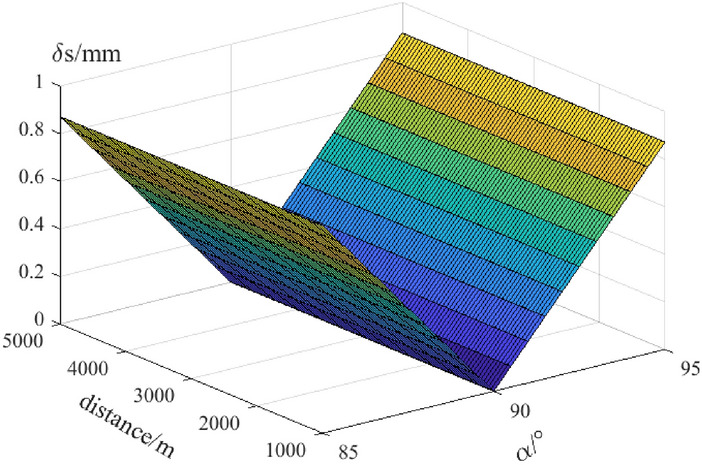
Measuring river-crossing distance difference between GNSS and total station.

In Fig. 3, the large distances of several kilometers on either side of the river from instrument to target, e.g. T1 to Z2 and T2 to Z1, are measured by GNSS receiver (Fig. 5) in place of the total station. However, the baseline of GNSS between receivers on either side of the river does not overlap the slope line between total station and target (prism) on either side of the river due to the height difference of the GNSS and total station. In practice, in the river-crossing levelling 0bservation process, we firstly use the total station to measure the zenith angle. Then remove the total station and 5-prism on either side of the river and put the GNSS receiver on the tripods. In order to maintain the instrument height difference of total station and GNSS receiver in 5 mm, we use a measuring tape to measure the height of the total station and 5-prism before removing them and use GNSS connector with the screw threads to adjust receiver height to correspond to the height of total station and 5-prism. Compared with the several kilometers baseline, at the condition of the height difference of instrument in 5 mm, the measuring river-crossing distance difference using GNSS and total station respectively can be ignored as shown in Fig. 6.

Figure 6 shows the measuring distance difference (${\delta }_{s}$) gradually increases with the increasing measuring distance. And if the zenith angle value is approximately equal to 90°, the minimum of ${\delta }_{s}$ will get. When the measuring distance changes from 1 to 5 km, the value of measuring distance difference changes from 0.1 mm to 0.9 mm. That is the baseline length of GNSS in the large distance can completely replace the distance from total station center to prism center.

### Vertical angle measurement

**Figure 7:**
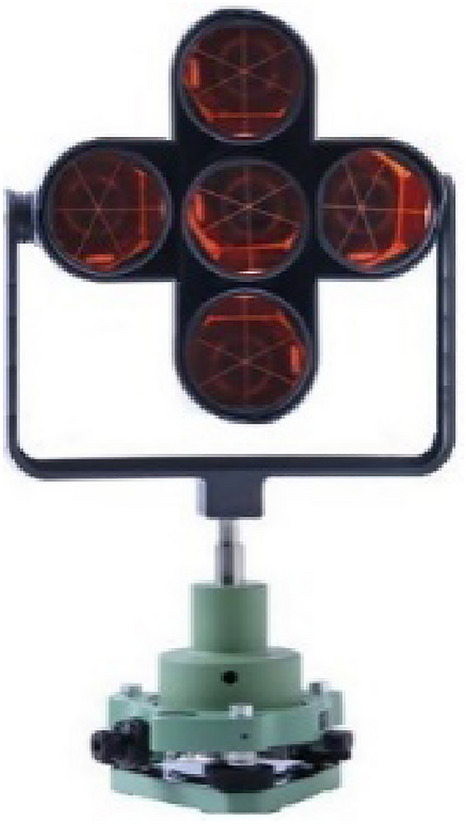
An integrated prism.

**Figure 8:**
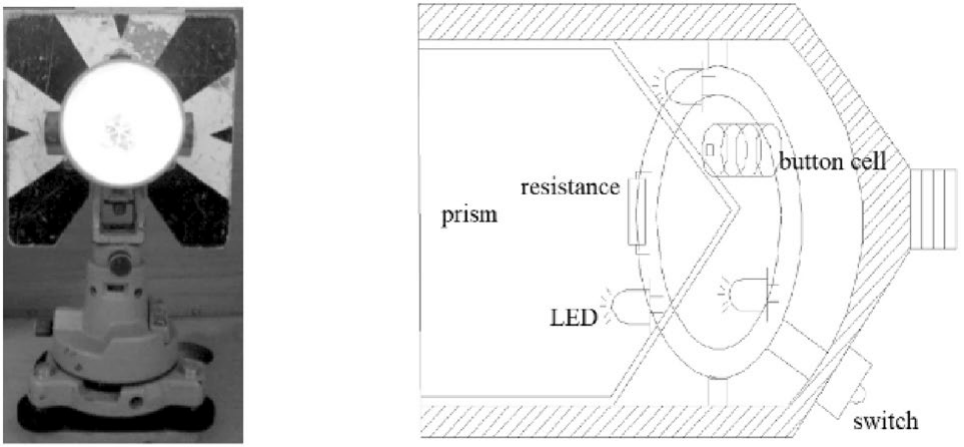
Prism with white LED.

In the early time of river-crossing levelling by the level instrument or total station, the pointing target is neither code bar rods nor prisms, but the large black and white plate^3,^^19,23^. And during the data collection, the surveyor with rich experience is needed to perform the observation. Therefore, it is not feasible to use a large plate as a pointing target in the long-distance river-crossing levelling. Considering the atmosphere refraction remains relatively stable in the evening and the wide width of the river is usually beyond the range of the automatic target recognition of robotic total station, this paper uses an integrated prism consisting of 5 prisms (named 5-prism; Fig. 7) and each prism has built-in LEDs (Fig. 8 (left)). And 5-prism has the advantage of small size and lightweight and is suitable for optical target recognition in the remote distance in the evening. Figure 8 (right) shows the structure of the inner of a prism with three LED lights installed. This circuit has a battery and a resistance and three LED lights. The voltage of a battery is 4.5 V and the resistance of a resistor is 65 Ω and the three lights are connected in parallel in the circuit.

As we all know, the principle of ATR of total station is adding the offset, calculated by the position of the center of the reflected laser spot in a pixel in COMS multiplies the angular resolution of one pixel corresponding, to the raw readings of zenith angle for the final accurate zenith angle. Therefore, to achieve the optical target recognition, firstly calculate position of the 5-prism spot center in image in pixels pictured by image-assisted total station. Then determine the angular resolution of one pixel corresponding. At last, based on the principle of ATR, the corrected zenith angle is the sum of offset and the raw reading.

Here, we take the telescope camera built in the Leica TM50 I total station as the COMS and take the 5-prism as the target. Leica TM50 I total station is equipped with two types of cameras, one is wild-angle camera and the other is a telescope camera. And both cameras support the GeoCOM command development and can set the image resolution, magnification and field of view (FOV). The specific parameters of cameras are shown in Table 1. From Table 1, the FOV of the camera is only related to the magnification of the picture no matter what the resolution of the picture is. Moreover, the FOV of a camera decreases with the increased magnification of the picture and the value of FOV is fixed at a certain magnification of the image.

**Table 1:** Parameters of telescope camera built in Leica TM50 I.

Picture format	Image resolution	Magnification	Horizontal FOV (deg)	Vertical FOV (deg)
.jpeg	2560 × 1920/1280 × 960/640 × 480/320 × 240	1 time	1.4146	1.0610
		2 times	0.7164	0.5373
		4 times	0.3582	0.2687
		8 times	0.1768	0.1326

**Figure 9:**
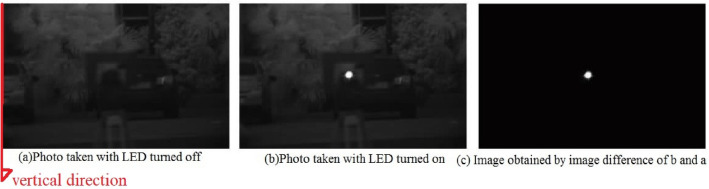
Extraction of LED spot by image difference method.

In order to extract the center of 5-prism spot’s position, many algorithms are compared including center of mass, template least squares matching and squared weighting grey centroid methods^15,24^. In practice, the grey value of spot is not uniform but like gaussian distribution and the grey value in the center of spot is higher than the edge of spot. Therefore, we select the squared weighting grey centroid method [formula (Eq. 13)] to locate the center of the spot because the method introduces the weight into the grey value. Besides, the surroundings of 5-prism are sometimes complex and other luminous targets will disturb the recognition of the 5-prism spot. In order to quickly extract the sketch of the 5-prism spot, we use the image difference method which means the subtraction of the grey values of two images (Fig. 9).

Because the telescope camera of Leica TM50 I total station has the fixed resolution of the image and fixed field of view at a certain magnification (Table 1). Therefore, one pixel corresponding to an angular resolution in the vertical direction of the photo is calculated as formula (Eq. 14).

13 $$y_{p} = \frac{{\sum\limits_{x = 1}^{m} {\sum\limits_{y = 1}^{n} {y \cdot f^{2} \left( {x,y} \right)} } }}{{\sum\limits_{x = 1}^{m} {\sum\limits_{y = 1}^{n} {f^{2} \left( {x,y} \right)} } }}$$

where $y_{p}$ denotes the vertical pixel coordinate of spot center,$f\left( {x,y} \right)$ denotes the pixel grey value, $\left( {x,y} \right)$ denotes the pixel coordinate, $m \times n$ denotes image resolution.

14 $$\Delta \beta = \frac{F}{n}$$

where $\mathrm{n}$ denotes vertical photographic resolution, $\mathrm{F}$ denotes the vertical field of view.

According to formula Eqs. (13) and (14), the zenith angle of accurate aiming is as follows:

15 $$\beta = (\frac{n}{2} - y_{p} ) \cdot \Delta \beta + \beta_{p}$$

where $\upbeta$ denotes zenith angle representing the aiming LED spot center, ${\upbeta }_{p}$ denotes raw zenith angle when image-assisted total station takes a photo.

**Figure 10:**
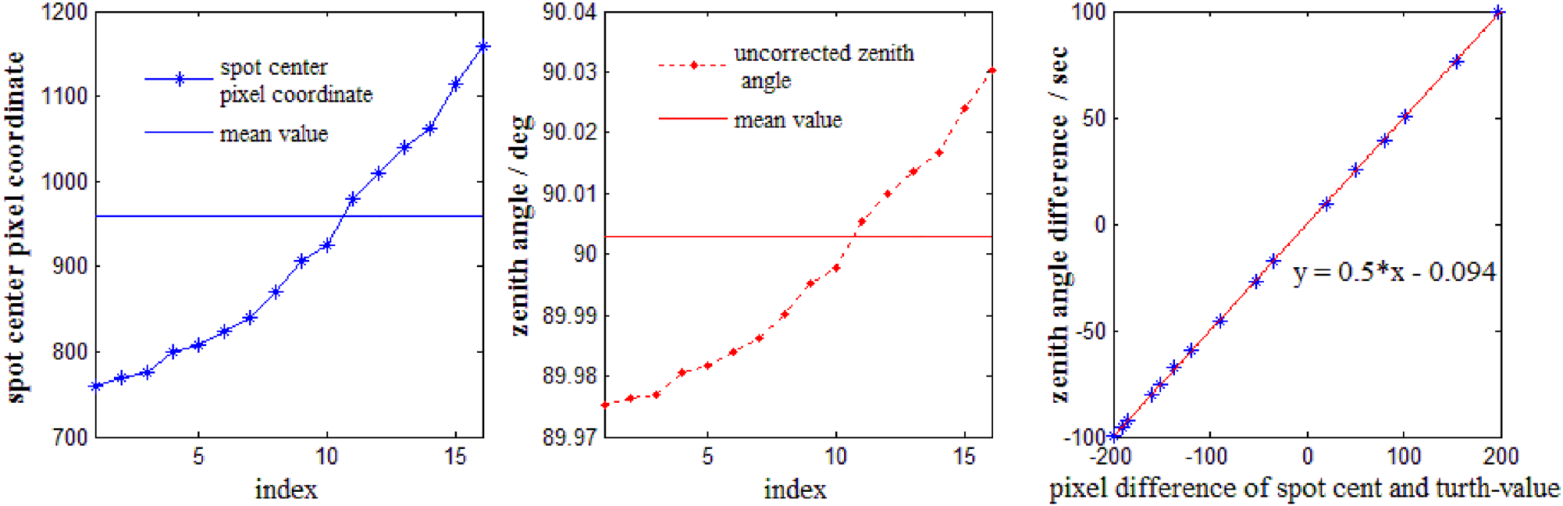
Test of LED recognition and aiming by Leica TM50 I image-assisted total station at 1 km.

Here take Leica TM50 I total station (series 371472) as an example, the camera vertical resolution $\mathrm{n}$ is 1920 and its field of view in the vertical direction is 0.2687 degrees at 4 times magnification, so $\Delta\upbeta$ is 0.5 s. In this experiment, all the zenith angle readings are collected at the face left of total station. The true zenith angle is 90°0′10″ obtained by total station ATR mode at the distance of 1 km. The zenith angle readings and spot center coordinates at a different place by adjusting the vertical circle of total station and are shown in Fig. 10 (left and middle). The linear fitting of pixel difference and zenith angle difference is described as shown in Fig. 10 (right). The linear fitting formula is in accord with the value of $\Delta\upbeta$. However, the mean value of the corrected zenith angle is 90°00′16″, the mean value of the corrected spot vertical center coordinate is 970.13. Compared with truth-values of zenith angle and camera center coordinate, the difference of corrected value and truth-value are 6″ and 10.13 pixels respectively. Therefore, multiple observation sets are needed in practice to ensure the accuracy of the target aiming.

## Application

**Figure 11:**
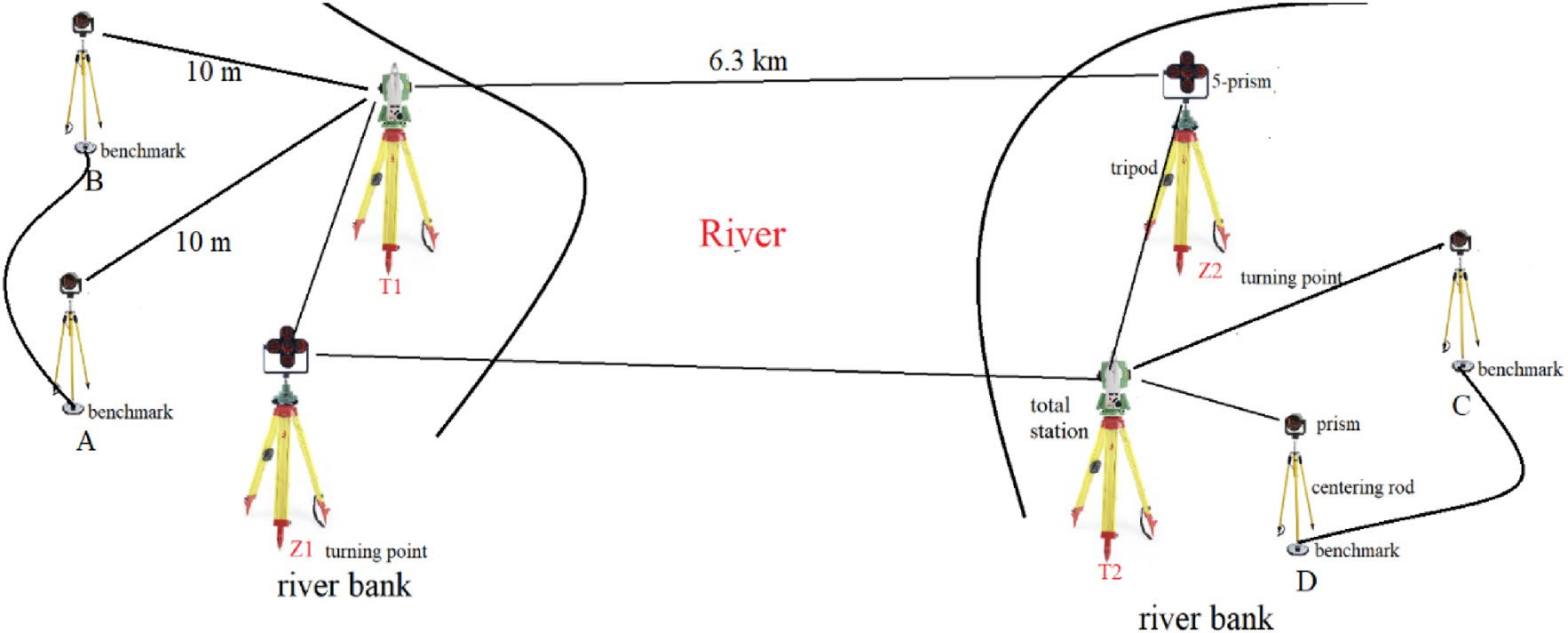
Observation route of Fuzhou Bridge in China. Leica TM50 I total station and GPR1 circle prism are used and the observation in conducted in the evening.

Here takes the rive-crossing levelling in Fuzhou Bridge in China as an example (Fig. 11). The topography of two sides of the river is similar and the altitudes of two sides are approximate. There are two benchmarks on either side of the bank and the distance of two benchmarks on the same side of the riverbank is ~ 10 m. The height differences between benchmark A and B, C and D are all determined by the precise levelling with Trimble DiNi03 level instrument. And the levelling line of benchmarks on the same side of riverbank is round trip observation. The distance across the river is about 6.3 km and the measuring procedure of river-crossing trigonometric levelling is as follows.

Step 1: Determine the height difference of total station and benchmark on the same side of the bank, e.g. ${h}_{T1,B}$, ${h}_{T1,A}$, ${h}_{T2,C}$ and ${h}_{T2,D}$. Set the total station measuring mode to ATR and input the pressure, temperature, and humidity for distance correction. The zenith angle observation and slope distance are measured in two observation sets. The measurements obtained on the face left of total station and that of face right are called one observation set.

Step 2: Measure the zenith angle between the image-assisted total station on one bank and a 5-prism on the other. And the LED built in the 5-prism turns on. As the river is too wide and the refraction effect is obvious, the zenith angle from T1 to Z2 (${\alpha }_{T1,Z2}$) and that from T2 and Z1 (${\alpha }_{T2,Z1}$) are performed simultaneously for reducing the refraction effect. The zenith angle observations were carried out in 2 periods and 8 groups in each period, and 8 observation sets were observed in each group. That is the total observation sets of a zenith angle is 128. When the total station records the raw zenith angles and the image of 5-prism spot is captured by total station synchronously. All the data is stored in the SD card of total station.

Then calculate the standard deviation and average of all observation sets of one zenith angle (${\alpha }_{T1,Z2}$ or ${\alpha }_{T2,Z1}$) and remove the observations beyond the 2 times of the standard deviation. If the amount of the remaining observations are more than two-thirds of total observations, the average of the remaining observations is taken as the final zenith angle. If not, add new observations and compute again until meeting the requirement mentioned above. Besides, to avoid the influence of system error of total station, the two total stations were interchanged and then the same process of zenith angle observation mentioned above was used for reverse measurement.

Step 3: Measuring distance across the river using GNSS. Remove the total station and 5-prism on the points of T1, Z1, T2 and Z2 and put the GNSS receivers on the points of T1, Z1, T2 and Z2. The TOPCONNET-G3A GNSS receivers whose nominal precise is 3 mm + 0.5 ppm are used to constitute a GNSS network (Fig. 5) and the GNSS network is continuously observed for 23.5 h and the lengths of baselines of T1T2, T1Z2, T2Z1 and Z2Z1 are obtained. The cut-off angle is set as 15° and the sampling interval is 30 s. Adopt the free network adjustment and the result of baselines of the network is shown in Table 2.

**Table 2:** Results of calculation of baseline.

Baseline	Length of baseline (m)	Standard deviation (m)
T1T2	6292.350	0.0001
T1Z2	6293.616	0.0001
T2Z1	6295.550	0.0001
Z2Z1	6297.004	0.0001

Here Table 3 shows the height difference of sides and closed loops in part different measuring groups and Table 4 shows the results of sides’ height difference after least square adjustment respectively.

**Table 3:** Height difference in partly different groups of observation side and closed loop.

Side (loop)	Height difference (m)									
AB	0.0147	0.0150	0.0152	0.0152	0.0149	0.0153	0.0149	0.0152	0.0150	0.0152
AD	− 0.2308	− 0.2334	− 0.2347	− 0.2269	− 0.2264	− 0.2239	− 0.2293	− 0.2295	− 0.2293	− 0.2295
AC	− 0.2318	− 0.2292	− 0.2261	− 0.2358	− 0.2127	− 0.2316	− 0.2250	− 0.2308	− 0.2218	− 0.2235
BD	− 0.2579	− 0.2406	− 0.2464	− 0.2405	− 0.2464	− 0.2438	− 0.2443	− 0.2475	− 0.2460	− 0.2458
BC	− 0.2432	− 0.2354	− 0.2396	− 0.2383	− 0.2429	− 0.2390	− 0.2361	− 0.2433	− 0.2396	− 0.2390
CD	0.0051	0.0053	0.0054	0.0049	0.0046	0.0054	0.0049	0.0053	0.0050	0.0052
ABD	− 0.0124	0.0079	0.0035	0.0016	− 0.0051	− 0.0046	− 0.0001	− 0.0028	− 0.0017	− 0.0011
ABC	0.0034	0.0088	0.0018	0.0128	− 0.0153	0.0078	0.0038	0.0027	− 0.0028	− 0.0003
ABCD	− 0.0027	0.0078	0.0049	− 0.0010	− 0.0062	− 0.0052	0.0032	− 0.0039	0.0097	0.0109

**Table 4:** Results of adjusted height difference.

Side	Height difference (m)	Distance (km)	Correction (mm)	Adjusted height difference (m)	Standard deviation/mm
AB	0.0151	0.010	0.00	0.0150	0.08
AD	− 0.2294	6.301	− 1.02	− 0.23042	0.58
AC	− 0.2268	6.303	1.47	− 0.22533	0.58
BD	− 0.2459	6.304	0.38	− 0.24552	0.58
BC	− 0.2396	6.305	− 0.83	− 0.24043	0.58
CD	0.0051	0.010	0.00	0.00510	0.08

The standard deviation for per kilometer:$\sqrt{\frac{\left[pvv\right]}{n-t}}=\pm 0.76 \mathrm{mm}.$

Table 3 shows the height difference in observation sides or closed loops in partly different observation groups. And the max difference of observation side’s height difference in different groups is 23.1 mm that is the AC side. And this value is less than the second-order levelling tolerance ${d}_{H}$ (${d}_{H}=4{M}_{\Delta }\sqrt{NS}=4\times 1\times \sqrt{8\times 6.3}=28.4 \mathrm{mm}$, where S is the length of river-crossing in kilometers, ${M}_{\Delta }$ is the mean square error of per kilometer and that of second-order levelling is 1 mm, N is the number of observation groups). The max value of closed-loop height difference is 9.9 mm that is an average of different groups of ABCD closed loop. And this value is less than closure loop tolerance $\mathrm{W}$ ($\mathrm{W}=6{M}_{w}\sqrt{S}$=6 $\times 2\times \sqrt{6.3}=30.1 \mathrm{mm}$, where ${M}_{w}$ is total mean square error and that of the second-order levelling is 2 mm).

Table 4 shows the results of each side’s height difference after adjustment. The max root-mean-square error of the observation side’s height difference is 0.58 mm and the accident mean square error of per kilometer is $\pm 0.76\mathrm{ mm}$, which are all less than the tolerance of second-order levelling.

## Discussion

Based on the principle of simultaneous reciprocal trigonometric levelling, we combined the image-assisted total station and GNSS for zenith angle observation and slope distance measurement respectively. The method we proposed extended the survey range of trigonometric levelling in the application of the river-crossing levelling and its precision met second order levelling. The high accuracy of our method mainly depended on the accuracy of baseline calculation using GNSS and remote target recognition using sub-pixel image processing technology.

During the process of the data collection, the difficulty of our method is the zenith angle observation. And the accuracy of zenith angle measurement mainly affects the accuracy of river crossing levelling. We use the 5-prism with LED lights installed as the target and the sub-pixel image processing algorithm to obtain the center of the 5-prism so that the accuracy of the zenith angle from the total station center to the 5-prism center is ensured. However, the random error is common during the zenith angle measurement. So, the average of multi observation sets of zenith angle is necessary as the final zenith angle observation. To ensure the accuracy of our method, we construct a survey routine consisting of closed loops. The height difference of a closed loop is measured by geometric levelling for short distance on the same bank and our method for long distance across the river. So that the result obtained by our method is verified by precise geometric levelling.

However, the pixel of the center of the 5-prism extraction is the critical factor to influence the accuracy of zenith angle measurement in our method. Moreover, this method we proposed is suitable for night as the illuminated target could be easy to recognize. Therefore, the more accurate and robust algorithm of extracting a 5-prism center is the future investigation. And the target which is adapt for day and night remains to be searched.

## Conclusion

High precision and efficiency height difference determination in river-crossing bridge construction are crucial. This paper analyses the errors of trigonometric levelling and proposes the precise trigonometric levelling method that combines the image-assisted total station and GNSS for river-crossing levelling at a long distance. The modular LED 5-prism is made as target and image processing and multi observation sets are adopted for automatic target recognition and aiming. Therefore, long-distance precise river-crossing trigonometric levelling can be realized based on those. In the practice of 6.3 km river-crossing trigonometric levelling, the standard deviation of the result is $\pm 0.76\mathrm{mm}/\sqrt{\mathrm{km}}$ that is compatible with the requirement of the second order precise levelling.
